# Handheld ECG Tracking of in-hOspital Atrial Fibrillation The HECTO-AF trial Clinical Study Protocol

**DOI:** 10.1186/s13063-019-3189-7

**Published:** 2019-01-30

**Authors:** Sara Schukraft, Marco Mancinetti, Daniel Hayoz, Yannick Faucherre, Stéphane Cook, Diego Arroyo, Serban Puricel

**Affiliations:** 1 Department of Cardiology, University and Hospital of Fribourg, Chemin des Pensionnats 2, 1708 Fribourg, Switzerland; 2 Department of General Internal Medicine, University and Hospital of Fribourg, Chemin des Pensionnats 2, 1708 Fribourg, Switzerland

**Keywords:** Atrial fibrillation, Atrial fibrillation screening, ECG handheld device, General medicine

## Abstract

**Background/rationale:**

Atrial fibrillation (AF) is frequent and causes great morbidity in the aging population. While initial events may be symptomatic, many patients have silent AF and are at risk of ischemic embolic complications. Timely detection of asymptomatic patients is paramount. The HECTO-AF trial aims to investigate the efficacy of an electrocardiogram (ECG) handheld device for the detection of AF in patients in hospital without a prior diagnosis of AF.

**Methods/design:**

The “Handheld ECG tracking of in-hospital atrial fibrillation” (HECTO-AF) study is a single-center, open-label, randomized controlled trial. The study population consists of all adult patients admitted to a general medicine ward of the University and Hospital of Fribourg throughout the study period. The study will enroll 1600 patients with 1:1 ratio allocation to either the detection group with one-lead handheld ECG recordings twice daily and extra recordings in the case of palpitations, versus a control group undergoing detection of AF as per routine clinical practice. Recordings will be self-performed after dedicated training, and will be independently adjudicated through a specific web-based interface. All enrolled patients will be followed clinically at 1, 2 and 5 years to assess the occurrence of AF, death, non-fatal stroke, systemic embolism, myocardial infarction and bleeding. The primary outcome is incidence of newly detected AF during the hospital stay. Secondary outcomes are incidence of AF, cardiovascular death, stroke, myocardial infarction and bleeding complications at 1, 2 and 5 years.

**Discussion:**

HECTO-AF is an independent randomized study aiming to detect the incidence of silent AF in all-comers hospitalized in general medicine wards.

**Trial registration:**

ClinicalTrials.gov, NCT03197090. Registered on 23 June 2017. Local ethical Committee (CER-VD) registration number: 2017–01594. There are no conflicts of interest to declare.

**Electronic supplementary material:**

The online version of this article (10.1186/s13063-019-3189-7) contains supplementary material, which is available to authorized users.

## Background

### Background and rationale

Atrial fibrillation (AF) is the most common arrhythmia [1, 2], affecting an estimated 3% of the general population above 20 years old [3]. The prevalence has risen over past decades due to shifting demographics resulting in an older population [4]. The prevalence of in-hospital AF is 10–31% [5–7]. Patients with AF have increased morbidity and a twofold increase in mortality when compared with the general population [8], and it is estimated that one third of patients with AF will be hospitalized at least once a year due to worsening heart failure, cardioembolic events or progressive dementia [9]. Thus, AF negatively impacts public health.

When symptomatic, AF may induce palpitations, chest pain, fatigue, and exercise intolerance. However, at least a third of patients with AF are asymptomatic. Furthermore, only 20% of symptomatic patients with AF will have symptoms temporally related to their AF episodes [10]. For this reason, the prevalence of AF may be much higher than has been previously reported. The EMBRACE [11] and CRYSTAL-AF trials [12] demonstrated that prolonged electrocardiogram (ECG) monitoring significantly improved detection of AF in patients with cryptogenic stroke or transient ischemic attack (TIA). Recognizing silent AF in the general population, and subsequent treatment could reduce morbidity and mortality [13].

The European Society of Cardiology (ESC) guidelines recommend AF screening by palpation or resting ECG in all patients > 65 years of age, and especially in populations at increased risk [14]. Recently, single-lead ECG recording devices have been developed, allowing quick, precise and non-invasive detection of AF.

The present study aims to determine the incidence of silent AF using a single-lead ECG handheld device in patients hospitalized in the department of general internal medicine of the University and Hospital of Fribourg, Switzerland. Participants will be randomized to either the intervention screening group with twice-daily single-lead ECG recordings and 12-lead ECG in the case of symptoms or to the comparator control group consisting of standard care.

## Methods

### Participants, interventions, and outcomes

#### Study setting

**Fig. 1:**
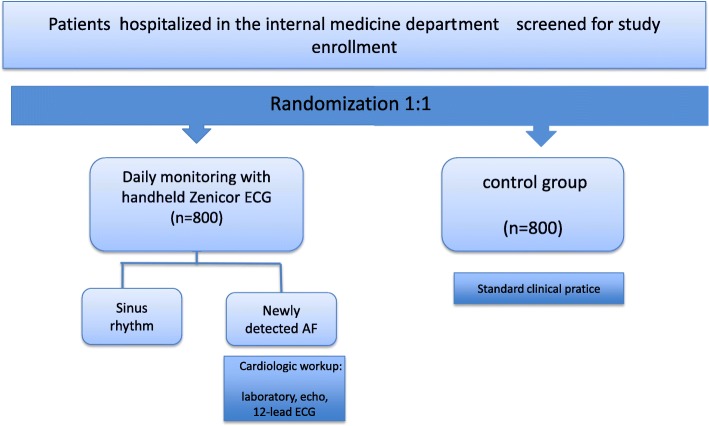
Study flowchart. Participants are randomized 1:1 to either handheld single-lead electrocardiogram (ECG) (Zenicor) screening or the control group

This is a single-center, open-label, randomized controlled trial with an allocation ratio of 1:1 conducted in the University and Hospital of Fribourg, Switzerland. All adult patients admitted to the General Internal Medicine Department will be screened for study enrollment. The Standard Protocol Items: Recommendation for Interventional Trials (SPIRIT) checklist can be found in Additional file 1 and the SPIRIT figure is included in the main body of the manuscript (Fig. 1).

#### Eligibility criteria

All patients 18 years or older admitted to the General Internal Medicine Ward are eligible. Patients with known or previously documented AF, patients with cardiac pacemakers or implantable cardioverter-defibrillators and those unable or unwilling to provide written informed consent will be excluded.

#### Interventions

Patients allocated to the intervention group will undergo single-lead handheld ECG recordings using the Zenicor device (https://zenicor.com/), twice daily and extra recordings in the case of palpitations during the period of hospitalization. Recordings will be self-performed after dedicated training and according to the Zenicor user manual.

**Fig. 2:**
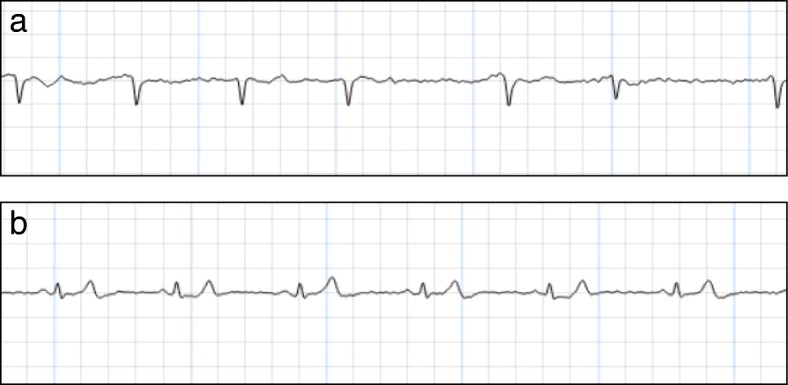
Electrocardiogram (ECG) recording with Zenicor, showing atrial fibrillation (AF) (**a**) and sinus rhythm (**b**)

The thumbs of the patient are placed onto the device during 30 s to yield a single-lead ECG recording, which is stored and subsequently transmitted to a central server for clinical analysis. The device has been shown to have higher sensitivity for detection of AF than the conventional 24-h Holter device (sensitivity (SN) 96%, specificity (SP) 92%) [15]. The Zenicor ECG device was chosen for ease of use and reliability, after a test phase in our outpatient clinic including two other devices: the KardiaMobile (https://www.alivecor.com/) and the ME90 mobile ECG (https://www.beurer.com/web/gb/products/medical/ecg-and-pulse-oximeter/mobile-ecg-device/). Examples of recordings are depicted in Fig. 2. The trial follows the instruction of the European Directive on medical devices 93/42/EEC and the ISO Norm 14,155 and ISO 14971, the Swiss Law and Swiss regulatory authority requirements. A specially trained nurse will check for appropriate recording after measurement of routine vital parameters during daily rounds.

All recordings will be independently adjudicated through a specific Web-based interface. Patients allocated to the control group will undergo detection as per routine clinical practice (i.e. symptom-based, in the case of suspicion on auscultation or during measurement of vital parameters if prescribed). The only foreseeable discontinuation criterion in either group would be participant request. Compliance in the intervention group is ensured by the presence of a dedicated nurse and twice-daily Web-based checks for ECG compliance by study investigators.

#### Patients with new onset of AF

Each patient with newly diagnosed AF will be treated according to the 2016 ESC guidelines on the management of AF (i.e. on anticoagulation, rhythm and/or rate control therapy). Additional workup including 12-lead ECG, laboratory investigations and further cardiological workup such as echocardiography, will be organized as per guidelines.

#### Outcomes

The primary outcome is the incidence of new-onset AF defined as a 30-s recording of irregular rhythm without p waves during hospitalization [14]. Two independent cardiologists will examine each case of newly diagnosed AF using the Zenicor device. In the case of uncertainty, additional recordings such as 12-lead ECG, 24-h Holter recording or 7-day R-Test may be required.

The secondary outcomes are the incidence of ischemic stroke, systemic embolism, cardiovascular death and myocardial infarction during hospitalization and at 1, 2 and 5 years. Secondary endpoints will be assessed by a telephone interview. If the patient is unavailable, the data will be retrieved from the referring physician/general practitioner, the hospital database or family/relatives of the patient. Local cantonal death registries will be consulted if the information is otherwise unavailable.

#### Participant timeline

**Fig. 3:**
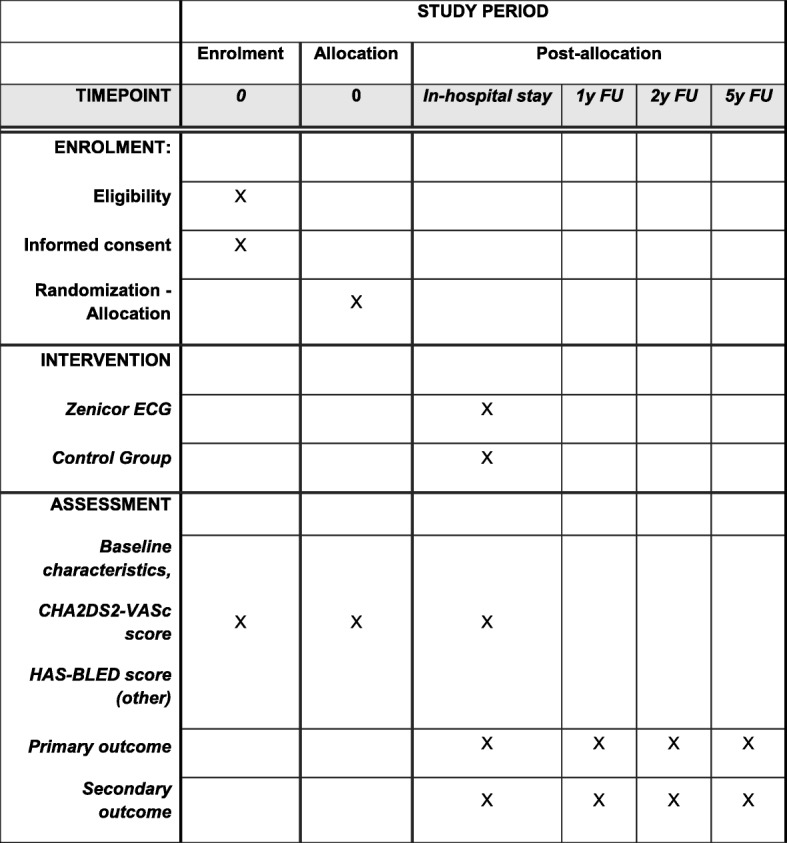
Timetable of investigations, interventions and assessments. ECG, electrocardiogram; FU, follow up; y, year. Primary outcome: new-onset atrial fibrillation (AF). Secondary outcome: incidence of ischemic stroke or systemic embolism, myocardial infarction, cardiovascular death or heart failure

The timeline for enrollment, interventions, and investigations is outlined in Fig. 3.

#### Sample size

Based on previous research, we estimate to detect AF in 1% of patients in the control group and in 3% of patients in the intervention group. The inclusion of 745 patients per group (total 1490) would yield power of 80% to detect this 2% difference between groups. We seek to include 800 patients per group (total 1600) to account for ineffective measurements, post-inclusion dropouts or other unforeseen dropouts.

#### Recruitment

Recruitment will be carried out by one full-time study nurse. All patients admitted to the General Internal Medicine Department at the Hospital of Fribourg will be screened. If the eligibility criteria are met the patient will be formally enrolled. All other nurses and physicians in the department have been informed of the study and trained in the use of handheld ECG devices.

### Assignment of interventions

#### Allocation

The randomization sequence was generated via computer (randomizer.org). Patients are randomly assigned in a 1:1 ratio to single-lead Zenicor ECG recording or standard care. The randomization list will be sealed in opaque envelopes and kept by a secretary not involved in the study to guarantee allocation blinding.

#### Blinding

There is no blinding or masking in the study. The statistician is the only individual blinded to treatment allocation.

### Data collection, management and analysis

#### Data collection methods

The following baseline characteristics will be collected by the study nurse from the patient’s medical records: patient demographics (age, gender, body mass index) and concomitant diseases including but not limited to hypertension, dyslipidemia, diabetes mellitus, heart failure, vascular disease, previous stroke/thromboembolism, renal failure, chronic respiratory disease and other endocrine anomalies (e.g. thyroid disease). Data on other variables will be collected, including symptoms such as palpitations, chest pain, the CHA2DS2-VASc clinical prediction score for risk of stroke and the HAS-BLED score for risk of major bleeding, diagnosis for index hospitalization and medical treatment. Characteristics of screened patients excluded from or unwilling to participate in the trial will not be collected. Selection bias may arise because these patients might differ from the study population with regard to the aforementioned characteristics, which in turn would represent a major study limitation.

#### Data management

Patient characteristics, study outcomes and clinical follow up will be recorded in an electronic case report form (eCRF; REDcap Software, Vanderbilt University, Nashville, TN, USA [16]). A trained study nurse will complete data management procedures, including coding (through de-identified numbers), verification, validation, security and storage of the database. The investigators will have access to the protocol, dataset and statistical code during and after the study for publication and dissemination. The study nurse will only have access to the dataset during the study time. During the clinical trial, data will be accurately recorded and the original documents will be stored at the clinical trials unit of the University and Hospital of Fribourg, under locked conditions when not in use. At the end of the study, Zenicor ECG recordings will be saved in PDF form and stored for 10 years. The server is in an ISO27001-certified secure data center. The data collected will not include identifiable references to the subjects. The confidentiality of records will be protected.

#### Statistical methods

We plan to enroll 800 patients in each group. Sample size calculations were performed using STATA (StataCorp. 2013. Stata Statistical Software: Release 13. College Station, TX, USA: StataCorp LP). Based on previous figures, the expected annual detection rate is 3% in the Zenicor group and 1% in the control group. The inclusion of 745 patients per group would yield power of 80% at a two-tailed significance level of α = 0.05. To account for ineffective measurements, post-inclusion dropouts or other unforeseen dropouts, we seek to include 800 patients per group. Categorical variables will be reported as counts and percentages. Continuous variables are presented as mean ± standard deviation or median (25–75%) interquartile range according to their distribution. Normality will be assessed by visual inspection of histograms, the computation of Q-Q plots and the Shapiro-Wilk test. Categorical variables will be compared using the chi-square or Fisher’s exact test as appropriate. Continuous variables will be analyzed using Student’s *t* test or the Wilcoxon rank-sum test according to the data distribution. Survival free from the occurrence of the clinical end points will be compared using the log-rank test and plotted as Kaplan-Meier survival functions. All statistical analyses will be performed using dedicated software (Stata version 15, StataCorp LP, College Station, TX, USA: StataCorp LP) at a two-tailed significance level of α = 0.05. The null hypothesis to be rejected is that there is no difference in the percentage of patients with newly detected AF in the control and the intervention group. The primary analysis pertains to the proportion patients with newly detected AF. Univariate methods (chi-square test) will be employed. If a significant difference in baseline characteristics is observed, then logistic regression will be performed to correct for baseline imbalances.

A secondary analysis will focus on subgroups. Pre-specified subgroups comprise gender, age, diabetes mellitus, hypertension, ischemic heart disease, congestive heart failure and all subcategories of the CHA2DS2-VASc score. Additional analysis will be performed on clinical follow up. Event-free survival in the intervention and the control group will be compared at 1, 2 and 5 years using the Kaplan-Meier method. Adjustment for imbalances will be performed using Cox proportional hazards given that the data do not violate the proportionality assumption.

Missing data will be omitted pairwise from the analyses. Whenever data are missing, this will be explicitly stated in the final results of the study. Missing data arising from loss to follow up will be censored. The number of dropouts will be provided in a flow-chart along with the reasons for the dropouts.

#### Monitoring

This is a monitoring study with no additional risks to the patients so no external data monitoring committee is required. The principal investigator and a review board composed of the statistician and the study nurse will perform ongoing monitoring. An interim analysis is planned after inclusion of the first 800 patients for quality management; there will be no early discontinuation of the trial.

#### Harms

No harm is to be expected from the intervention itself. However, active screening for AF can lead to both false positive or false negative results. This may lead to unwarranted anticoagulant treatment, exposing the patient to an increased risk of bleeding. Conversely, in misdiagnosed patients it may give a false sense of security and increased risk of stroke in patients with high CHA2DS2-VASc scores. To minimize this, as previously mentioned, two independent cardiologists will review every case of new AF diagnosed by the Zenicor device. In the case of uncertainty, additional recordings, e.g. 12-lead ECG, 24-h Holter ECG or 7-day R-Test may be performed.

## Ethics and dissemination

### Research ethics approval

This study is conducted in compliance with the current version of the Declaration of Helsinki. The research project was approved by the local ethics committee of Vaud, Switzerland (CER-VD 2017–01594).

### Protocol amendments

The principal investigator is responsible for communicating important protocol modifications to the ethics committee and to the competent authorities including the clinical trial registry (ClinicalTrials.gov).

### Consent

The study nurse will collect patient information at day 1 and provide a consent form with details on trial rationale, interventions, potential benefits and harms. The patients will be given up to 24 h to consider participation. Once having provided written informed consent, the patient will be enrolled and randomized to one of the treatment groups. Patients can withdraw their consent unconditionally, at any time and without any justification. Medical data that have been collected to date will, however, be analyzed.

### Confidentiality

The investigators will comply with local privacy laws. Anonymity of the participants will be guaranteed when presenting the data at scientific meetings or publishing them in scientific journals. Individual subject medical information obtained as a result of this study is considered confidential and disclosure to third parties is prohibited.

### Access to data

Data will be stored physically and electronically on the personal computers at the clinical trials unit at the University and Hospital of Fribourg. Physical data are protected by restricted access to their location. Electronic data are protected by the IT-Services of the state of Fribourg (SITEL services). The investigators will have access to the protocol, dataset, and statistical code during and after the study for publication and dissemination. The study nurse will only have access to the dataset during the study period.

### Declaration of interest

The trial is supported by an unrestricted grant from the Fonds Scientifique Cardiovasculaire Fribourg.

### Dissemination policy

The study results will be disseminated within the department of General Internal Medicine, and are intended to be published in peer-reviewed medical journals and communicated at medical conferences.

## Discussion

Silent AF is common [17], especially in older patients and those with heart failure [18], and may lead to stroke or death [19, 20]. The 2016 ESC guidelines encourage systematic AF screening programs in at-risk populations [14], which include most patients admitted to general medicine wards, who are older and have several comorbidities. It has been shown that the CHADS2 and CHA2DS2-VASc scores are directly associated with the incidence of new-onset AF and reliably predict AF [21]. A recent study assessing the performance of handheld ECG devices to detect AF in cardiology and geriatric wards [22] found acceptable sensitivity (81–89%) and specificity (94–97%) compared to standard ECG.

The Zenicor ECG is a well-validated device that is non-invasive and easy to use. The product has been used regularly since 2010 in over 350 clinics in Europe. Sensitivity and specificity compared to 12-lead ECG are 96% and 92%, respectively [23]. The device has been evaluated for AF screening and has shown higher sensitivity for detection of silent AF compared to conventional 24-h Holter recordings [24, 25]. One of the advantages compared to other devices includes direct mobile-network transmission to a Web-based central database for rapid online analysis.

A recent Cochrane review on systematic AF screening suggests that future studies should examine the effectiveness of alternative screening strategies such as handheld devices [26]. As previously mentioned, timely detection of silent AF could have major benefits to patients by decreasing both morbidity and mortality. Whether or not this is cost-effective and acceptable to a healthcare network still needs to be determined. The HECTO-AF trial will assess the use of a handheld single-lead ECG device for the screening of silent AF in patients admitted to a general medicine ward.

### Trial status

Recruitment started on 1 March 2018; the planned completion date is 30 March 2019.

## Additional file

Additional file 1: SPIRIT 2013 checklist. (DOC 121 kb)

## Abbreviations

AF: Atrial fibrillation
CER-VD: Ethics committee of Vaud
CRYSTAL-AF: Cryptogenic stroke and underlying atrial fibrillation
ECG: Electrocardiogram
EMBRACE: 30-Day cardiac event monitor belt for recording atrial fibrillation after a cerebral ischemic event
ESC: European Society of Cardiology
HECTO-AF: Handheld ECG tracking of in-hospital atrial fibrillation
R-test: Electrocardiogram event recorder
SITEL: Services of the state of Fribourg
SN: Sensitivity
SP: Specificity
TIA: Transient ischemic attack
